# Acute effects of doorway stretch on the glenohumeral rotational range of motion and scapular position in high-school baseball players

**DOI:** 10.1016/j.jseint.2021.07.002

**Published:** 2021-08-28

**Authors:** Takashi Higuchi, Yuichi Nakao, Yasuaki Tanaka, Masashi Sadakiyo, Koki Hamada, Shigeki Yokoyama

**Affiliations:** a Department of Physical Therapy, Osaka University of Human Sciences, Shojaku, Settsu-city, Osaka, Japan; b Department of Rehabilitation, Furukawa Miyata Orthopedic and Internal Medicine Clinic, Nagasaki-city, Nagasaki, Japan; c Department of Rehabilitation, Saiseikai Nagasaki Hospital, Nagasaki-city, Nagasaki, Japan; d Department of Rehabilitation, Sadamatsu Hospital, Omura-city, Nagasaki, Japan; e Department of Physical Therapy, Kyoto Tachibana University, Kyoto-city, Kyoto, Japan

**Keywords:** Baseball, Doorway stretch, Pectoralis minor, Glenohumeral internal rotation, Scapular position, GIRD

## Abstract

**Background:**

Pectoralis minor tightness has been thought to affect the scapular position. Despite the wide implementation of doorway stretch in clinical practice owing to its apparent effectiveness in stretching the pectoralis minor, limited studies have investigated its influence on the glenohumeral rotational range of motion (ROM). This study aimed to examine the acute effects of doorway stretch on the glenohumeral rotational ROM and the correlation between the scapular position and ROM.

**Materials and Methods:**

In total, 34 male high-school baseball players participated in this study. Outcomes included the pectoralis minor muscle length, glenohumeral rotational ROM, and scapular position. The distance between the sternocostal joint of rib 4 and the coracoid process was measured as the pectoralis minor length. Internal and external rotation ROM with the shoulder abducted at 90° were measured. The scapular position was defined by the following two measurements: the distance between the surface and the posterolateral corner of the acromion as well as the scapular index. The participants performed doorway stretch by abducting and externally rotating the shoulder at 90° and flexing the elbow at 90°. The outcomes were compared before and after stretching. Furthermore, the correlation between these changes was investigated.

**Results:**

Pectoralis minor muscle length and glenohumeral internal rotation ROM was significantly higher after stretching than before, and the scapular position also significantly changed (*P* < .01 for both). A moderate correlation was found between the degree of change in the glenohumeral internal rotational ROM and scapular position (r = 0.44, *P* < .01).

**Discussion:**

Our results indicated that doorway stretch significantly increased the pectoralis minor muscle length and glenohumeral internal rotation ROM and significantly altered the scapular position. Furthermore, a significant correlation between the degree of change in the scapular position and glenohumeral internal rotation ROM was observed. These results suggest that doorway stretch could be beneficial for improving the scapular position and glenohumeral internal rotation ROM, which are considered the cause of throwing injuries. Furthermore, the glenohumeral ROM may be affected by the scapular position.

Limited glenohumeral internal rotation range of motion (ROM) among overhead athletes—otherwise known as glenohumeral internal rotation deficit (GIRD)^9^—has been identified as a cause of throwing injuries. GIRD has been considered the cause of anterior humeral head translation during motion,^12^^,^^25^ which leads to glenohumeral pathologies such as superior labral anterior and posterior lesions or internal impingements.^8^^,^^9^^,^^27^^,^^31^ Some researchers have shown that posterior shoulder tightness (PST) is one of the causes of GIRD.^32^^,^^41^ Therefore, posterior shoulder stretching has been widely used to resolve PST.^22^^,^^35^ Laudner et al.^20^ reported that the acute effects of the sleeper stretch do not sufficiently promote clinical changes despite being statistically significant. This result suggests that to restore PST, stretching alone does not always effectively improve GIRD.

The scapular position has been considered to be another factor affecting glenohumeral internal rotation ROM. Borich et al^3^ reported a correlation between the scapular anterior tilt angle in static posture and the glenohumeral internal rotation ROM. In addition, Ribeiro and Pascoal^33^ reported that overhead athletes had more scapular anterior tilt in the dominant side when performing active internal rotation of the glenohumeral joint with the arm abducted. Taken together, the aforementioned findings show that the scapular position may affect the glenohumeral internal rotation ROM.

Tightness of the pectoralis minor has been thought to affect the scapular position. The pectoralis minor originates from ribs 3–5 and inserts into the coracoid process (CP), enabling protraction, downward rotation, and depression of the scapula.^13^ Borstad and Ludewig^7^ reported that doorway stretch effectively stretched the pectoralis minor, leading to scapular retraction with the shoulder in the abducted position. Although doorway stretch has been widely implemented in clinical practice, limited studies have investigated its influence on glenohumeral ROM. This study, therefore, aimed to examine the acute effects of doorway stretch on glenohumeral rotational ROM.

## Materials and methods

### Participants

Participants who played baseball at a high school were recruited using personal contacts. The inclusion criteria were male baseball players and a baseball-playing experience of >3 years. The exclusion criteria were a history of shoulder or elbow surgery and clear deformities of the scapula or thorax. Outcomes included the pectoralis minor muscle length, glenohumeral rotational ROM, acromial distance, and scapular index indicating the scapular position. Approval was obtained from the ethics committee of the Kibi International University in Okayama, Japan, and all participants and their guardians provided written and verbal informed consent.

### Measurements

#### Pectoralis minor muscle length

Pectoralis minor muscle length was measured as the distance from the sternocostal joint of rib 4 (Rib 4) to the CP (Rib 4–CP distance).^4^ The Rib 4 and CP were identified through palpation, after which an 8-mm-diameter tack seal was attached on the skin at these landmarks. Identification of the Rib 4 first required identifying the first sternocostal joint through palpation. Thereafter, by descending along the joints one by one, the Rib 4 was identified. The CP was also identified through palpation, after which a tack seal was attached at the most prominent area. The distance between the tack seals, which indicated the pectoralis minor muscle length, was measured using a tape measure to 1-mm precision.^5^ When the measurements were taken, the marked sites were palpated and confirmed through the tack seal again to minimize skin movement. Participants were instructed to sit on a chair without a back rest and maintain a natural, but not rounded, posture with their upper extremities relaxed. Furthermore, participants were instructed to avoid taking deep breaths and to stare at a point in front of them to avoid neck movement and postural change. Measurements were taken once by a physiotherapist with at least 5 years of experience who was blinded to the participant information. The test-retest reliability was assessed previously. Accordingly, 17 healthy shoulders were measured thrice within 1 day; the intraclass correlation coefficient (ICC 1,3) and standard error of mean (SEM) values were 0.93 and 0.2 cm, respectively.

#### Glenohumeral ROM

Glenohumeral ROM was measured with the participants in the supine position with their arms abducted at 90°, elbow flexed at 90°, and their forearm placed in a neutral position. An investigator then grasped the participant’s shoulder with their thumb on the CP and the other four fingers on the posterior aspect of the scapula while the other hand grasped the participant’s wrist and passively rotated it internally and externally. Another investigator was responsible for attaching the digital inclinometer (Shinwa Rules, Sanjo, Japan) with a precision of 0.1° to the participant’s forearm to measure the glenohumeral abduction internal rotation angle (ABIR) and abduction external rotation angle (ABER). This measurement was performed once bilaterally. Two physiotherapists with >5 years of experience who were blinded to the participant information performed the measurements. Total motion arc (TMA) was defined as the sum of the ABIR and ABER. GIRD was calculated by subtracting the dominant ABIR from the nondominant ABIR. The ICC and SEM of the ABIR were 0.98 and 3.9°, respectively, whereas those of the ABER were 0.97 and 4.4°, respectively.

#### Scapular position

##### Acromial distance

Participants were positioned in the supine position on a rigid surface with their arms at the side of their body and their forearms in a neutral position. The acromial distance was measured as the distance between the surface and the posterolateral corner of the acromion (PLA)^39^ using a digital vernier caliper (Shinwa Rules, Sanjo, Japan) with 0.1-mm precision. Measurements were taken in the following two conditions: supine with static position (AD-S) and supine with scapular retraction position (AD-R). The ICC and SEM of the AD-S were 0.98 and 4.0 cm, respectively, and those of the AD-R were 0.97 and 3.4 cm, respectively.

##### Scapular index

With participants in a neutral sitting position on a chair without a back rest, an investigator measured the distance from the participant’s sternal notch (SN) to the CP (SN–CP distance). Thereafter, the distance from the PLA to the thoracic spine (TS) at the same level (PLA–TS distance) was measured. The scapular index, which has been thought to indicate the scapular position reflecting the effect of pectoralis minor tightness,^4^ was calculated as follows: scapular index = (SN−CP distance/PLA−TS distance) × 100. The ICC and SEM were 0.94 and 0.7, respectively. Both the acromial distance and scapular index were measured by a physiotherapist with >5 years of experience who was blinded to the participant information.

### Intervention

**Figure 1:**
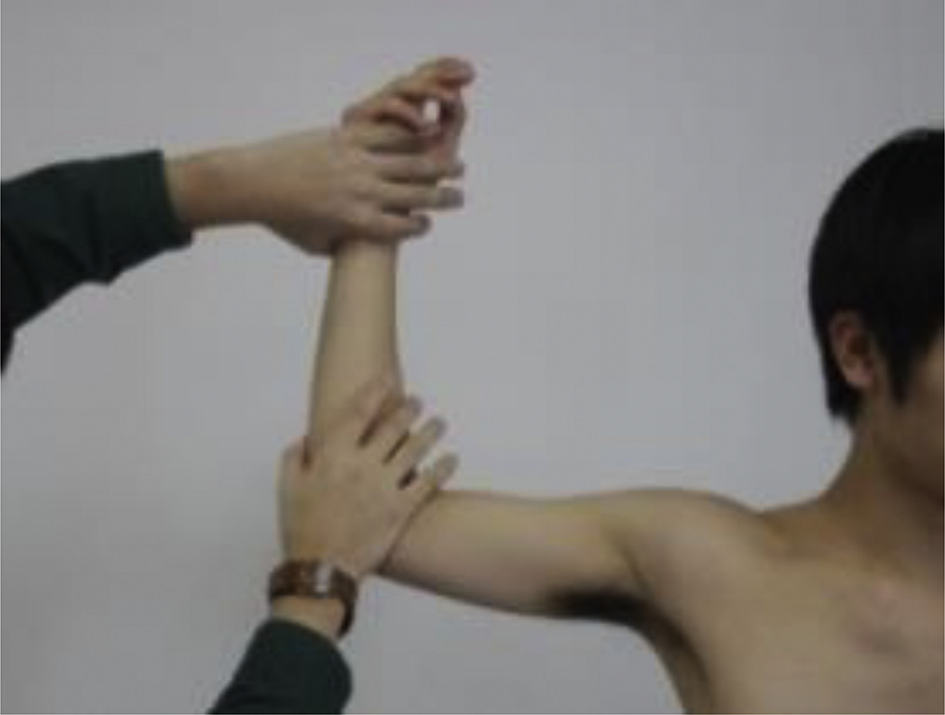
Doorway stretch. The participant’s forearm is stabilized by an investigator.

The participants performed doorway stretch unilaterally on their dominant arm by abducting and externally rotating the shoulder at 90° and flexing the elbow at 90° (Fig. 1). An investigator fixed the participant’s elbow and wrist in front of them. Thereafter, the participants rotated their trunk to the opposite side until they could maximally stretch their chest without experiencing any pain.^7^ Participants performing doorway stretch were instructed to retract the scapula and avoid having their shoulder protrude forward to prevent glenohumeral horizontal abduction. Participants held this position for 30 seconds and repeated the stretching 5 times, with a 10-second rest between the repetitions where the arms were placed in a relaxed position.^1^^,^^23^ The same investigator calculated the equation for the stretching effect.

### Statistical analysis

An investigator who was blinded to all measurements performed the statistical analysis. A paired *t*-test was used to determine prestretch and poststretch differences, and the correlation between each degree of change was analyzed using the Pearson correlation coefficient. SPSS (version 20.0; IBM Inc., Armonk, NY, USA) was used for statistical analysis, with an alpha value of 5% indicating statistical significance.

## Results

**Figure 2:**
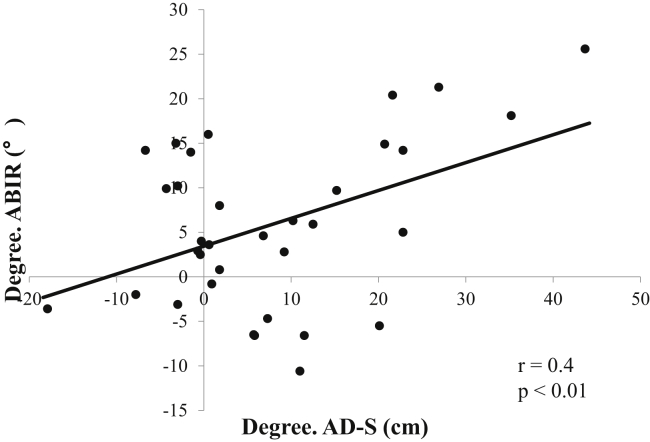
Significant correlation between the degree of change in internal rotation range of motion and that in acromial distance in the static position (r = 0.44; *P* < .01). ABIR, glenohumeral internal rotation angle with the shoulder abducted at 90°; AD-S, acromial distance in the static position.

In total, 34 male high-school baseball players were recruited for this study (Table I). Compared with the outcome data before stretching, the Rib4–CP distance, ABIR, TMA, and scapular index significantly increased after stretching; however, the AD-S and AD-R significantly decreased after stretching (Table II). Regarding the acromial distance, the degree of change in both the AD-S and AD-R significantly correlated with that in GIRD (Table III). Moreover, the degree of change in the AD-S significantly correlated with that in the ABIR (Fig. 2).

**Table I:** Participant demographics.

Variables	n = 34	
	Mean ± SD	Range
Age (yr)	16.4 ± 0.6	16-17
Height (cm)	171.2 ± 6	160-184
Weight (kg)	67.9 ± 8.3	53-85
Exposure (yr)∗	8.4 ± 2.3	4-11

*SD*, standard deviation.

∗ Participant self-report.

**Table II:** Comparison between each variable before and after stretching.

Variables	Pre	Post	*P* value	Cohen's d	95% CI for Cohen's d	
					Lower	Upper
Pectoralis minor length (cm)						
Dominant Rib4–CP	16.1 ± 1.2	16.7 ± 1.1	**<.01**	−0.32	−0.8	−0.2
Nondominant Rib4–CP	16.7 ± 1.0	16.8 ± 1.0	**<.01**	−0.09	−0.2	−0.05
GH ROM (°)						
Dominant ABIR	42.9 ± 10.9	48.7 ± 12.0	**<.01**	−0.54	−8.8	−2.4
Nondominant ABIR	57.4 ± 10.6	59.6 ± 9.5	.41	−0.23	−3.9	1.7
Dominant ABER	103.0 ± 7.7	102.0 ± 8.8	.6	0.03	−2.4	2
Nondominant ABER	95.2 ± 9.3	93.7 ± 6.2	.26	0.16	−1.2	4.4
Dominant TMA	145.9 ± 13.3	150.9 ± 15.7	**.03**	−0.37	−9.1	−1.1
Nondominant TMA	153.3 ± 15.1	153.9 ± 10.6	.82	−0.1	−5.1	4
GIRD	14.7 ± 13.7	11.0 ± 13.1	.11	0.41	0.6	9.9
Scapular position						
Dominant AD-S (mm)	70.3 ± 19.9	62.3 ± 14.0	**<.01**	0.29	3.4	13.8
Nondominant AD-S (mm)	64.8 ± 15.7	65.0 ± 15.7	.94	−0.02	2.1	11.6
Dominant AD-R (mm)	46.2 ± 16.0	37.7 ± 11.3	**<.01**	0.13	4.7	10.7
Nondominant AD-R (mm)	37.8 ± 10.7	39.5 ± 11.7	.54	−0.15	1.1	7.6
Dominant scapular index	64.7 ± 4.2	67.4 ± 5.3	**<.01**	−0.72	−4.4	−1.6
Nondominant scapular index	69.3 ± 4.2	70.2 ± 4.3	**.03**	−0.32	−1.7	−0.1

*Pre*, before stretching; *Post*, after stretching; *95% CI*, 95% confidence interval; *Rib 4–CP*, distance from the sternocostal joint of rib 4 to the coracoid process; *GH ROM*, glenohumeral range of motion; *ABIR*, glenohumeral internal rotation angle with the shoulder abducted at 90°; *ABER*, glenohumeral external rotation angle with the shoulder abducted at 90°; *TMA*, total motion arc; *GIRD*, glenohumeral internal rotation deficit; *AD-S*, acromial distance in the static position; *AD-R*, acromial distance with the scapula retracted; *SD*, standard deviation.

Data are expressed as mean ± SD.

Bold font indicates statistical significance.

**Table III:** Spearman correlation matrix between each degree of change in variables.

Variables	Degree	Degree	Degree	Degree	Degree	Degree	Degree
	Rib4–CP	ABIR	ABER	TMA	GIRD	AD-S	AD-R
Degree. ABIR	−0.07						
Degree. ABER	−0.04	−0.05					
Degree. TMA	−0.08	**0.67**∗	**0.71**∗				
Degree. GIRD	0.01	**0.76**∗	−0.22	**0.38**†			
Degree. AD-S	−0.15	**0.44**∗	−0.18	0.17	**0.46**∗		
Degree. AD-R	−0.06	0.31	−0.39	0.19	**0.40**†	**0.63**∗	
Degree. Scapular index	**0.63**∗	−0.04	−0.23	−0.21	0.01	−0.08	0.08

*Degree*, degree of change; *Rib 4–CP*, distance from the sternocostal joint of rib 4 to the coracoid process; *ABIR*, glenohumeral internal rotation angle with the shoulder abducted at 90°; *ABER*, glenohumeral external rotation angle with the shoulder abducted at 90°; *TMA*, total motion arc; *GIRD*, glenohumeral internal rotation deficit; *AD-S*, acromial distance in the static position; *AD-R*, acromial distance with the scapula retracted.

Bold font indicates statistical significance.

∗ *P* < .01.

† *P* < .05.

## Discussion

This study revealed that performing doorway stretch increased the pectoralis minor muscle length, improved scapular position, and increased glenohumeral rotational ROM. Furthermore, a significant correlation was observed between the degree of change in the scapular position and that in the ABIR.

In this study, the scapular index significantly increased after the intervention, whereas the acromial distance significantly decreased. These results indicate that the scapular position was more posteriorly tilted and was further externally rotated after doorway stretch than before it. The asymmetry of the scapular position between the dominant and nondominant sides is quite common in overhead athletes.^11^^,^^30^^,^^42^ However, this asymmetry is one of the potential causes of throwing injuries.^8^^,^^17^ Burkhart et al^8^ advocated the use of “SICK scapula”; this acronym stands for Scapular malposition, Inferior medial border prominence, Coracoid pain and malposition, and dysKinesis. They theorized this was related to the throwing injuries. Tightness in the pectoralis minor is believed to be one of the causes of scapular malposition.^4^^,^^6^^,^^14^ Umehara et al^44^ revealed that stretching with the shoulder horizontally abducted, which is a similar position to that of doorway stretch, is effective for stretching the pectoralis minor.^2^^,^^7^^,^^26^ Therefore, doorway stretch is thought to increase the extensibility of the pectoralis minor. Several researchers have reported that scapular manual retraction,^18^ repositioning,^40^ and assistance^36^ improve strength in the muscles surrounding the shoulder, alter the subacromial space associated with shoulder injury, and reduce shoulder pain. Therefore, doorway stretch is also thought to improve scapular position and be an effective treatment option for throwing injuries.

Our study findings indicated that the ABIR is increased as a result of performing doorway stretch. The maximum internal rotation of the angular velocity during throwing motion reaches 7000–8500° per second, and the shoulder uses >1000 N of proximal force to decelerate arm movement.^24^^,^^29^ Repetitive throwing motion leads to adaptive changes, such as increases in the ABER and GIRD.^22^^,^^24^^,^^37^^,^^46^ GIRD is thought to result from the thickness or stiffness in the posterior shoulder joint capsule or rotator cuff muscles,^27^^,^^41^ and it is generally referred to as PST. It is reported that PST can contribute to throwing injuries.^9^^,^^25^ Furthermore, Wilk et al^46^ proposed the TMA concept, and Kibler et al^19^ stated that total arc deficit can cause throwing injuries. Therefore, improving the ABIR is important for the treatment of throwing injuries. Several studies have examined the effects of the posterior shoulder stretch on GIRD improvement.^16^^,^^21^^,^^43^^,^^46^ McClure et al^23^ compared the effects of the cross-body stretch and the sleeper stretch in a randomized controlled trial and found that these increased the ABIR by 20° and 12°, respectively. On the other hand, Laudner et al^20^ reported that the sleeper stretch immediately increased the ABIR by only 3.1°, which was an insignificant clinical change. Muraki et al^26^ clarified in a cadaveric study that the ABIR is improved by 2° when the posterior capsule is elongated by a cyclic testing device. In this study, the degree of improvement in the ABIR was 5.8°, which is higher than the results of the study by Laudner et al.^20^ Stretching the posterior shoulder soft tissue is beneficial, but it is possible that this stretch is sometimes insufficient for improving the ABIR. Doorway stretch also appears to be beneficial for improving the ABIR in overhead athletes.

In this study, a significant correlation was observed between the degree of decrease in the AD-S and that in the ABIR. It has been reported that the scapular position and humeral head position are highly correlated.^45^ Ribeiro and Pascoal^33^ reported the correlation between the scapular anterior tilted position and GIRD. Kibler^17^ proposed that the scapula functioned as a stable base for the glenohumeral joint, which serves an important function for throwers. Changes in the scapular position, especially additional posterior tilt, and external rotation position may, thus, be considered to affect the glenohumeral ROM. Further research is warranted to elucidate the detailed mechanism of changes in the scapular position and increase in the ABIR.

The strengths of this study are as follows. First, the same investigators who were blinded to the participant information performed the measurements to minimize errors and maintain consistent intervention intensity throughout the study. Second, the assessor was blinded to all the measurement results. Third, none of the measurements in this study required specific or expensive tools such as a three-dimensional motion capture system for performing the measurements. Therefore, these results are applicable to daily clinical practice. Fourth, the reliability and validity of the acromial distance and pectoralis minor muscle length measurements have been previously established.^28^^,^^34^^,^^38^^,^^39^

This study also has some noteworthy limitations. First, this study did not include control participants. Nonetheless, this study aimed to examine the acute effects of doorway stretch and measure its outcomes immediately thereafter. Thus, very few effects besides the stretch effect were noted. Second, scapular position measurements were taken only in the resting position. Third, the reliability and validity of the scapular index have not been firmly established, although it has been used in several studies.^10^^,^^15^^,^^47^ Fourth, participants in this study were recruited from a high-school baseball team. However, they had a mean experience in baseball of 8 years. In general, Japanese high-school baseball players start their careers approximately at the age of 10 years. Therefore, the participants’ experience in playing baseball was considered quite compatible with that of other high-school baseball players.

## Conclusion

Doorway stretch, which aims to stretch the pectoralis minor, changed the scapular resting position and increased the ABIR. Moreover, positive correlations were found between the degree of change in the scapular posterior tilted position and that in the ABIR. These results suggest that doorway stretch improves the shoulder ABIR and scapular position in baseball players.

## Disclaimers

*Funding:* No funding was disclosed by the author(s).

*Conflicts of interest:* The authors, their immediate families, and any research foundation with which they are affiliated have not received any financial payments or other benefits from any commercial entity related to the subject of this article.

## References

[1] Bandy W.D., Irion J.M., Briggler M. (1997). The effect of time and frequency of static stretching on flexibility of the hamstring muscles. Phys Ther.

[2] Beazell J.R., Magrum E.M. (2003). Rehabilitation of head and neck injuries in the athlete. Clin Sports Med.

[3] Borich M.R., Bright J.M., Lorello D.J., Cieminski C.J., Buisman T., Ludewig P.M. (2006). Scapular angular positioning at end range internal rotation in cases of glenohumeral internal rotation deficit. J orthopaedic Sports Phys Ther.

[4] Borstad J.D. (2006). Resting position variables at the shoulder: evidence to support a posture-impairment association. Phys Ther.

[5] Borstad J.D. (2008). Measurement of pectoralis minor muscle length: validation and clinical application. J orthopaedic Sports Phys Ther.

[6] Borstad J.D., Ludewig P.M. (2005). The effect of long versus short pectoralis minor resting length on scapular kinematics in healthy individuals. J orthopaedic Sports Phys Ther.

[7] Borstad J.D., Ludewig P.M. (2006). Comparison of three stretches for the pectoralis minor muscle. J Shoulder Elbow Surg.

[8] Burkhart S., Morgan C., Kibler W. (2003). The disabled throwing shoulder: spectrum of pathology Part III: the SICK scapula, scapular dyskinesis, the kinetic chain, and rehabilitation. Arthroscopy.

[9] Burkhart S.S., Morgan C.D., Kibler W.B. (2003). The disabled throwing shoulder: spectrum of pathology Part I: pathoanatomy and biomechanics. Arthroscopy.

[10] Carvalho L., Aquino C.F., Souza T.R., Anjos M.T.S., Lima D.B.M., Fonseca S.T. (2019). Clinical measures related to forward shoulder posture: a reliability and correlational study. J Manipulative Physiol Ther.

[11] Cools A.M., Johansson F.R., Cambier D.C., Velde A.V., Palmans T., Witvrouw E.E. (2010). Descriptive profile of scapulothoracic position, strength and flexibility variables in adolescent elite tennis players. Br J Sports Med.

[12] Gates J.J., Gupta A., McGarry M.H., Tibone J.E., Lee T.Q. (2012). The effect of glenohumeral internal rotation deficit due to posterior capsular contracture on passive glenohumeral joint motion. Am J Sports Med.

[13] Halder A.M., Itoi E., An K.-N. (2000). Anatomy and biomechanics of the shoulder. Orthop Clin North Am.

[14] Hébert L.J., Moffet H., McFadyen B.J., Dionne C.E. (2002). Scapular behavior in shoulder impingement syndrome. Arch Phys Med Rehabil.

[15] Hodgins J.L., Rubenstein W., Kovacevic D., Padaki A., Jobin C.M., Ahmad C.S. (2017). Pectoralis minor contracture in throwing shoulders of Asymptomatic adolescent baseball players. Orthopaedic J Sports Med.

[16] Johansen R.L., Callis M., Potts J., Shall L.M. (1995). A modified internal rotation stretching technique for overhand and throwing athletes. J Orthopaedic Sports Phys Ther.

[17] Kibler W.B. (1998). The role of the scapula in athletic shoulder function. Am J Sports Med.

[18] Kibler W.B., Sciascia A., Dome D. (2006). Evaluation of apparent and absolute supraspinatus strength in patients with shoulder injury using the scapular retraction test. Am J Sports Med.

[19] Kibler W.B., Sciascia A., Thomas S.J. (2012). Glenohumeral internal rotation deficit: pathogenesis and response to acute throwing. Sports Med Arthrosc Rev.

[20] Laudner K.G., Sipes R.C., Wilson J.T. (2008). The acute effects of sleeper stretches on shoulder range of motion. J Athletic Train.

[21] Maenhout A., Dhooge F., Van Herzeele M., Palmans T., Cools A. (2015). Acromiohumeral distance and 3-dimensional scapular position change after overhead muscle fatigue. J Athletic Train.

[22] Manske R., Wilk K.E., Davies G., Ellenbecker T., Reinold M. (2013). Glenohumeral motion deficits: friend or foe?. Int J Sports Phys Ther.

[23] McClure P., Balaicuis J., Heiland D., Broersma M.E., Thorndike C.K., Wood A. (2007). A randomized controlled comparison of stretching procedures for posterior shoulder tightness. J Orthopaedic Sports Phys Ther.

[24] Meister K. (2000). Injuries to the shoulder in the throwing athlete. Part one: biomechanics/pathophysiology/classification of injury. Am J Sports Med.

[25] Mihata T., Gates J., McGarry M.H., Neo M., Lee T.Q. (2015). Effect of posterior shoulder tightness on internal impingement in a cadaveric model of throwing. Knee Surg Sports Traumatol Arthrosc.

[26] Muraki T., Yamamoto N., Berglund L.J., Sperling J.W., Steinmann S.P., Cofield R.H. (2011). The effect of cyclic loading simulating oscillatory joint mobilization on the posterior capsule of the glenohumeral joint: a cadaveric study. J orthopaedic Sports Phys Ther.

[27] Myers J.B., Laudner K.G., Pasquale M.R., Bradley J.P., Lephart S.M. (2006). Glenohumeral range of motion deficits and posterior shoulder tightness in throwers with pathologic internal impingement. Am J Sports Med.

[28] Nijs J., Roussel N., Vermeulen K., Souvereyns G. (2005). Scapular positioning in patients with shoulder pain: a study examining the reliability and clinical importance of 3 clinical tests. Arch Phys Med Rehabil.

[29] Oi T., Yoshiya S., Slowik J., Diffendaffer A., Takagi Y., Tanaka H. (2019). Biomechanical differences between Japanese and American professional baseball Pitchers. Orthopaedic J Sports Med.

[30] Oyama S., Myers J.B., Wassinger C.A., Daniel Ricci R., Lephart S.M. (2008). Asymmetric resting scapular posture in healthy overhead athletes. J athletic Train.

[31] Paley K.J., Jobe F.W., Pink M.M., Kvitne R.S., ElAttrache N.S. (2000). Arthroscopic findings in the overhand throwing athlete: evidence for posterior internal impingement of the rotator cuff. Arthroscopy.

[32] Reinold M.M., Wilk K.E., Macrina L.C., Sheheane C., Dun S., Fleisig G.S. (2008). Changes in shoulder and elbow passive range of motion after pitching in professional baseball players. Am J Sports Med.

[33] Ribeiro A., Pascoal A.G. (2012). Scapular contribution for the end-range of shoulder axial rotation in overhead athletes. J Sports Sci Med.

[34] Rondeau M.W., Padua D.A., Thigpen C.A., Harrington S.E. (2012). Precision and validity of a clinical method for pectoral minor length Assessment in overhead-throwing athletes. Athletic Train Sports Health Care.

[35] Salamh P.A., Kolber M.J., Hanney W.J. (2015). Effect of scapular stabilization during horizontal adduction stretching on passive internal rotation and posterior shoulder tightness in young women volleyball athletes: a randomized controlled trial. Arch Phys Med Rehabil.

[36] Seitz A.L., McClure P.W., Finucane S., Ketchum J.M., Walsworth M.K., Boardman N.D. (2012). The scapular assistance test results in changes in scapular position and subacromial space but not rotator cuff strength in subacromial impingement. J Orthopaedic Sports Phys Ther.

[37] Shanley E., Thigpen C.A., Clark J.C., Wyland D.J., Hawkins R.J., Noonan T.J. (2012). Changes in passive range of motion and development of glenohumeral internal rotation deficit (GIRD) in the professional pitching shoulder between spring training in two consecutive years. J Shoulder Elbow Surg.

[38] Struyf F., Meeus M., Fransen E., Roussel N., Jansen N., Truijen S. (2014). Interrater and intrarater reliability of the pectoralis minor muscle length measurement in subjects with and without shoulder impingement symptoms. Man Ther.

[39] Struyf F., Nijs J., De Coninck K., Giunta M., Mottram S., Meeusen R. (2009). Clinical assessment of scapular positioning in musicians: an intertester reliability study. J Athl Train.

[40] Tate A.R., Mcclure P., Kareha S., Irwin D. (2008). Effect of the scapula reposition test on shoulder impingement symptoms and elevation strength in overhead athletes. J Orthopaedic Sports Phys Ther.

[41] Thomas S.J., Swanik C.B., Higginson J.S., Kaminski T.W., Swanik K.A., Bartolozzi A.R. (2011). A bilateral comparison of posterior capsule thickness and its correlation with glenohumeral range of motion and scapular upward rotation in collegiate baseball players. J Shoulder Elbow Surg.

[42] Thomas S.J., Swanik K.A., Swanik C.B., Kelly I.V.J.D. (2010). Internal rotation and scapular position differences: a comparison of collegiate and high school baseball players. J Athletic Train.

[43] Tyler T.F., Nicholas S.J., Roy T., Gleim G.W. (2000). Quantification of posterior capsule tightness and motion loss in patients with shoulder impingement. Am J Sports Med.

[44] Umehara J., Nakamura M., Fujita K., Kusano K., Nishishita S., Araki K. (2017). Shoulder horizontal abduction stretching effectively increases shear elastic modulus of pectoralis minor muscle. J Shoulder Elbow Surg.

[45] von Eisenhart-Rothe R., Matsen F.A., Eckstein F., Vogl T., Graichen H. (2005). Pathomechanics in atraumatic shoulder instability: scapular positioning correlates with humeral head centering. Clin Orthopaedics Relat Res.

[46] Wilk K.E., Meister K., Andrews J.R. (2002). Current concepts in the rehabilitation of the overhead throwing athlete. Am J Sports Med.

[47] Yuen G.K., Clements J.B., Ramalingam V., Sundar V. (2021). Understanding upper body playing-related musculoskeletal disorders among piano and non-piano players using a photogrammetry. La Clinica Terapeutica.

